# Urinary Metabolomics in Pediatric Obesity and NAFLD Identifies Metabolic Pathways/Metabolites Related to Dietary Habits and Gut-Liver Axis Perturbations

**DOI:** 10.3390/nu9050485

**Published:** 2017-05-11

**Authors:** Jacopo Troisi, Luca Pierri, Annamaria Landolfi, Francesca Marciano, Antonella Bisogno, Federica Belmonte, Carmen Palladino, Salvatore Guercio Nuzio, Pietro Campiglia, Pietro Vajro

**Affiliations:** 1 Department of Medicine, Surgery and Dentistry “Scuola Medica Salernitana”, Pediatric Section, University of Salerno, Via S. Allende, 84081 Baronissi (SA), Italy; luca.pierri@hotmail.com (L.P.); a.landolfi@hotmail.it (A.L.); francesca88.marciano@gmail.com (F.M.); a.bisogno29@studenti.unisa.it (A.B.); fecu91@gmail.com (F.B.); carmen.palladino@studenti.unina.it (C.P.); sguercio.nuzio@gmail.com (S.G.N.); pvajro@unisa.it (P.V.); 2 Theoreo srl, Via degli Ulivi 3, 84090 Montecorvino Pugliano (SA), Italy; 3 Department of Pharmacy, University of Salerno, Via G. Paolo II, 84084 Fisciano (SA), Italy; pcampiglia@unisa.it; 4 European Laboratory of Food Induced Disease (ELFID), 80100 Naples, Italy

**Keywords:** obesity, fatty liver, children, metabolome, urine

## Abstract

To get insight into still elusive pathomechanisms of pediatric obesity and non-alcoholic fatty liver disease (NAFLD) we explored the interplay among GC-MS studied urinary metabolomic signature, gut liver axis (GLA) abnormalities, and food preferences (Kid-Med). Intestinal permeability (IP), small intestinal bacterial overgrowth (SIBO), and homeostatic model assessment-insulin resistance were investigated in forty children (mean age 9.8 years) categorized as normal weight (NW) or obese (body mass index <85th or >95th percentile, respectively) ± ultrasonographic bright liver and hypertransaminasemia (NAFLD). SIBO was increased in all obese children (*p =* 0.0022), IP preferentially in those with NAFLD (*p =* 0.0002). The partial least-square discriminant analysis of urinary metabolome correctly allocated children based on their obesity, NAFLD, visceral fat, pathological IP and SIBO. Compared to NW, obese children had (1) higher levels of glucose/1-methylhistidine, the latter more markedly in NAFLD patients; and (2) lower levels of xylitol, phenyl acetic acid and hydroquinone, the latter especially in children without NAFLD. The metabolic pathways of BCAA and/or their metabolites correlated with excess of visceral fat centimeters (leucine/oxo-valerate), and more deranged IP and SIBO (valine metabolites). Urinary metabolome analysis contributes to define a metabolic fingerprint of pediatric obesity and related NAFLD, by identifying metabolic pathways/metabolites reflecting typical obesity dietary habits and GLA perturbations.

## 1. Introduction

The incidence of obesity and its related conditions, including non-alcoholic fatty liver disease (NAFLD), has dramatically increased worldwide in all age groups [1]. Given the health and economic burden and consequences of these conditions, their prevention and treatment have become major priorities. Current standard dietary and lifestyle changes, and pathogenetically oriented treatments often fail due to poor compliance and/or lack of efficacy [2]. Consequently, a better understanding of the pathomechanisms underlying obesity and NAFLD is necessary for more satisfactory and efficient therapeutic approaches.

Several lines of evidence indicate that specific gut microbiota may play a role in obesity, metabolic syndrome, and fatty liver by increasing energy harvesting in conditions of intestinal dysbiosis or small intestinal bacterial overgrowth (SIBO) [3,4]. In the presence of a damaged intestinal barrier (“leaky gut”), the gut-liver axis (GLA) may amplify he normal interactions between intestinal bacteria/bacterial products and hepatic receptors [5], thereby promoting a cascade of events, namely, oxidative stress, insulin-resistance (IR), hepatic inflammation and fibrosis, via a large number of metabolites [6].

Numerous previous studies found that urinary/blood high levels of aromatic (AAA) ± branched chain amino acids (BCAA) are associated with insulin resistance and the risk of obesity-related metabolic syndrome (MetS) [7,8,9]. It has been reported a link between BCAAs and IR due to interferences with insulin signaling [10]. Acylcarnitine catabolism ± changes in nucleotides, lysolipids and inflammation markers also appear to be implicated in obesity and its related disorders [11]. Based on these and other urine- and/or plasma-based studies of pediatric obesity and MetS [12,13,14], we hypothesized that metabolite profiles differences between lean and obese children with and without NAFLD [7] depend on/are in relation with GLA abnormalities, which is a novel concept in this area of study.

## 2. Materials and Methods

### 2.1. Subjects

Forty consecutive Italian children/adolescents (mean age 9.8 years) were enrolled in this pilot case-control study after receiving written informed consent from parents/guardians. Subjects were recruited at a teaching hospital and at a related health-service outpatient obesity clinic. The inclusion criteria were: age between 5 and 16 years, normal weight (BMI from the 25th to 85th percentile), obese (BMI > 95th percentile), and absence of acute intercurrent or chronic illness. The cohort study was carried out in accordance with the ethic principles of the declaration of Helsinki 2013 [15] and approved by the local institutional ethics committee.

#### 2.1.1. Anthropometric Measurements and Lifestyle Evaluation

For each child, we measured the weight, height, BMI values and percentiles, waist circumference (WC) percentiles as a measure of central (i.e., visceral) obesity, waist-to-height ratio, blood pressure and cardiac frequency. Anthropometric measurements were obtained by trained staff members using calibrated instruments and standardized methods. The BMI percentiles were evaluated on the basis of the Italian reference percentiles (2–20 years) for height, weight, and BMI [16]. Waist circumferences were evaluated based on European percentiles [17].

Questionnaires were administered to evaluate lifestyle including alcohol intake, and total daily caloric and nutrients intake as previously described [18]. The medical history included information on usual food preferences and was investigated by the validated KIDMED (Mediterranean Diet Quality Index) scale [19].

#### 2.1.2. Imaging Investigations and Biochemical Parameters

All children and controls underwent abdominal ultrasonography (US) to establish the presence/absence of bright liver (hepatic steatosis). Ultrasound examination was performed with an apparatus equipped with a convex pediatric probe (Aloka, Wallingford, CT, USA). Bright liver was assessed with standard ultrasound criteria [20,21]. Based on ultrasound results, subjects were divided in three groups: obese subjects without hepatic steatosis (*N =* 13), obese subjects with hepatic steatosis (*N =* 13), and age-matched, normal-weight healthy subjects (*N =* 14) (recruited in the pediatric surgery ward while attending for minor surgery). There were no significant differences between BMI classes of pre- and post-pubertal children. Since BMI reflects both adiposity and muscle mass, the grade of the children’s central obesity was evaluated based on the number of centimeters exceeding the 90th age and gender specific percentile of WC.

Intestinal barrier damage was diagnosed based on IP assessed by HPLC analysis of lactulose and mannitol urinary values 5 h after sugar ingestion in fasting children. Lactulose/mannitol ratios (L/M ratio) were considered abnormal when they exceeded 0.03 [22]. Small intestinal bacterial overgrowth was identified using a hydrogen breath test (H2BT) apparatus (Bedfont Scientific Ltd., Maidstone, Kent, UK). H_2_ basal values >40 ppm or an increase of 20 ppm over the baseline within the first 120 min were considered suggestive of SIBO [23]. Serum levels of glucose, insulin, alanine transaminase (ALT), aspartate transaminase (AST), and homeostatic model assessment-insulin resistance (HOMA-IR) were recorded. Children with ultrasonographic bright liver ± hypertransaminasemia [24] underwent transaminase retesting, creatine phosphokinase determination and laboratory exclusion of the most frequent causes of pediatric liver disease other than NAFLD (autoimmune hepatitis, Wilson disease, celiac disease, alpha1-anti trypsin deficiency, viral hepatitis A, B, and C, Cytomegalovirus, and Epstein Barr virus). Finally, only 22 patients with concordance of both US liver brightness and transaminase values data were considered as being affected (*N =* 12) or not affected (*N =* 10) by obesity-related NAFLD. Another urine sample was obtained and immediately stored at −20 °C until metabolome analysis.

### 2.2. Untargeted Metabolomics Analysis

#### 2.2.1. Metabolite Extraction and Derivatization

Metabolome extraction, purification and derivatization was carried with the MetaboPrep GC kit (Theoreo srl, Montecorvino Pugliano (SA), Italy) according to the manufacturer’s instruction.

#### 2.2.2. GC-MS Analysis

Two µL samples of the derivatized solution were injected into the GC-MS system (GC-2010 Plus gas chromatograph coupled to a 2010 Plus single quadrupole mass spectrometer; Shimadzu Corp., Kyoto, Japan). Chromatographic separation was achieved with a 30 m 0.25 mm CP-Sil 8 CB fused silica capillary GC column with 1.00 µm film thickness from Agilent (Agilent, J&W Scientific, Folsom, CA, USA), with helium as carrier gas. The initial oven temperature of 100 °C was maintained for 1 min and then raised by 4 °C/min to 320 °C with a further 4 min of hold time. The gas flow was set to obtain a constant linear velocity of 39 cm/s and the split flow was set at 1:5. The mass spectrometer was operated in electron impact (70 eV) in full scan mode in the interval of 35–600 *m/z* with a scan velocity of 3333 amu/s and a solvent cut time of 4.5 min. The complete GC programme duration was 60 min. Untargeted metabolites were identified by comparing the mass spectrum of each peak with the NIST library collection (NIST, Gaithersburg, MD, USA). Some of the over 250 signals per sample produced by gas chromatographic hyphenated mass spectrometry were not investigated further because they were not consistently found in other sets of samples (either too low in concentration or of poor spectral quality to be confirmed as metabolites). A total of 196 endogenous metabolites involved in energy metabolism, lipid metabolism and amino acid metabolism were detected sequentially. To identify peaks, the linear index difference max tolerance was set at 10, while the minimum matching for the NIST library search was set at 85%. Results were summarized in a comma-separate matrix file and loaded in the appropriate software for statistics manipulation. The chromatographic data for PLS-DA analysis were tabulated with one sample per row and one variable (metabolite) per column. The normalization procedures consisted of data transformation and scaling. Data transformation was made by generalized log transformation and data scaling by autoscaling (mean-centered and divided by standard deviation of each variable).

### 2.3. Statistical Analysis

#### 2.3.1. Monovariate Analysis

Statistical analysis was performed using Statistica software (StatSoft, Tulsa, OK, USA) and Minitab (Minitab Inc., State College, PA, USA). Normal distribution of data was verified using the Kolmogorov-Smirnov test. Since the data were normally distributed, we used one-way ANOVA with the Tukey post hoc test for inter-group comparisons. The alpha value was adjusted according to Bonferroni setting to 0.05/15 = 0.003.

#### 2.3.2. Multivariate Data Analysis

Partial least square discriminant analysis (PLS-DA) was performed on Internal Standard peak area [25] normalized chromatogram using R (Foundation for Statistical Computing, Vienna, Austria). Mean centering and unit variance scaling was applied for all analyses. Classes separation was archived by PLS-DA, which is a supervised method that uses multivariate regression techniques to extract, via linear combinations of original variables (X), the information that can predict class membership (Y). PLS regression was performed using the plsr function included in the R pls package [26]. Classification and cross-validation was performed using the corresponding wrapper function included in the caret package [27]. A permutation test was performed to assess the significance of class discrimination. In each permutation, a PLS-DA model was built between the data (X) and the permuted class labels (Y) using the optimal number of components determined by cross validation for the model based on the original class assignment. Two types of test statistics were used to measure class discrimination. The first is based on prediction accuracy during training. The second made use of separation distance based on the ratio between group sum of the squares and the Within group sum of squares (B/W-ratio). If the observed test statistics was part of the distribution based on the permuted class assignments, class discrimination cannot be considered significant from a statistical point of view [28]. Variable Importance in Projection (VIP) scores were calculated for each component. A VIP is a weighted sum of squares of the PLS loadings, taking into account the amount of explained Y-variation in each dimension.

The metabolic pathway was constructed using MetScape application [29] of the software Cytoscape [30]. To identify changes in metabolite levels associated with the three continuous variables, namely, the L/M ratio, centimeters exceeding the 90th percentile and KidMed Score, we carried out three stepwise multivariate linear regression analyses, with L/M, centimeters exceeding the WC 90th percentile and KidMed score as the dependent variables.

## 3. Results

The demographic and clinical–laboratory characteristics of cases and controls are reported in Table 1. None of the NW controls had biochemical/US hepato-metabolic abnormalities. Most obese children followed unhealthy diets, as reflected by KidMed scores significantly lower compared with their normal weight peers. Dietary nutrients assessment confirmed that their diets were significantly more caloric, richer in total and saturated fats, and (in obese with NAFLD) in fructose as well (Table S1).

More than fifty percent of obese children (*N =* 12) had US signs of NAFLD and hypertransaminasemia not due to the most common causes of liver diseases, and significantly higher values of systolic blood pressure (*p =* 0.0003) and glycaemia (*p =* 0.002). Obese NAFLD children had a higher frequency of SIBO and significantly altered IP.

**Figure 1:**
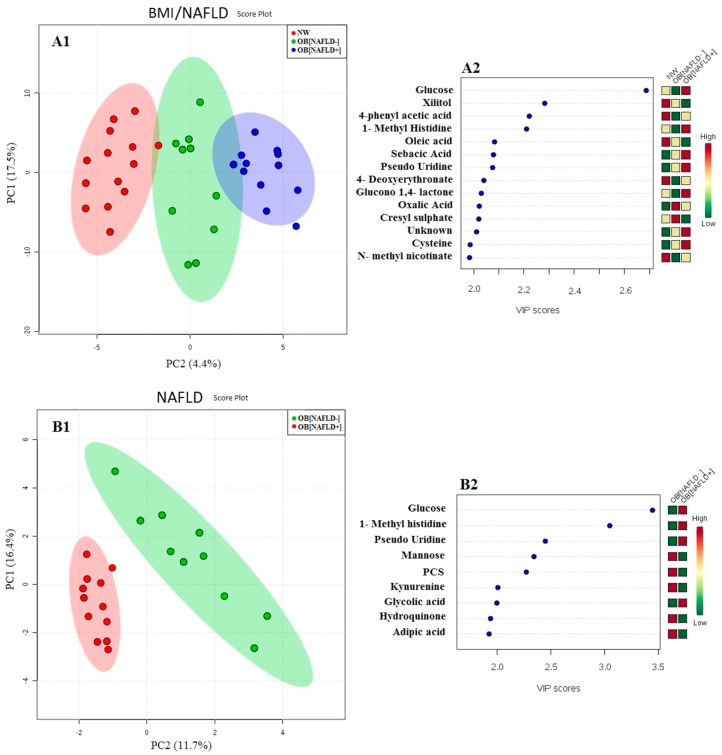

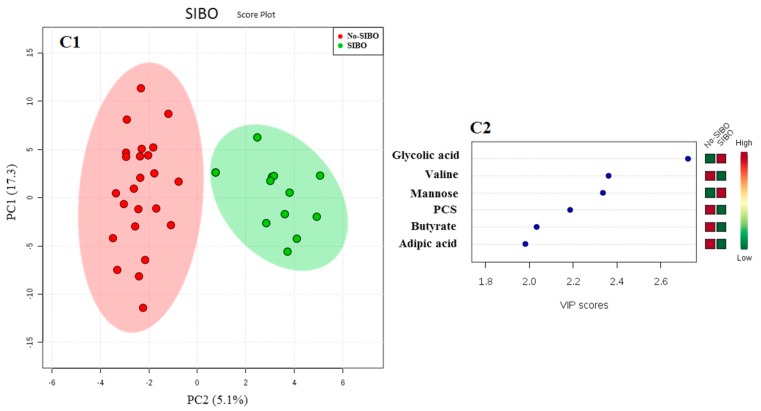
Partial least square discriminant analysis (PLS-DA) models to discriminate children according to Body Mass Index (BMI) ± Non-Alcoholic Fatty Liver Disease (NAFLD) (**A1**); NAFLD (**B1**), and Small Intestinal Bacterial Overgrowth (SIBO) (**C1**) as unique parameters investigated. The explained variance of each component is shown in brackets on the corresponding axis. In panel (**A1**), blue circles represent the obese children with NAFLD (OB[NAFLD+]), green circles represent obese children without NAFLD (OB[NAFLD−]), while red circles represent the normal weight controls (NW). In panel (**B1**), red ellipse contains NAFLD negative obese children, while green one the NAFLD positive obese children. In panel (**A3**), red ellipse contains SIBO negative children, while green the SIBO positive children. The first 14, 9, and 6 VIP variables identified by the corresponding PLS-DA are shown in Panels (**A2**), (**B2**) and (**C2**), respectively. The number of VIPs was established by setting the VIP-score ≥ 2 as a cut off value. In panel (**A2**) one VIP was not identified because its mass spectrum did not reach the predefined library comparison performance (in terms of similar index or Kovats index difference). In all cases, the colored boxes on the right indicate the relative amount of the corresponding metabolite in each group under study.

As shown in Figure 1A1, the PLS-DA score plots clearly differentiated among obese children with and without steatosis and NW controls. The 14 VIP variables identified by PLS-DA are shown in Figure 1A2. The number of VIPs was established by setting the VIP-score > 2 as a cut off value.

The PLS-DA model also discriminated NAFLD-positive from NAFLD-negative obese individuals without overlap (Figure 1B1). Nine metabolites have a VIP-score > 2 (Figure 1B2).

A third PLS-DA model (Figure 1C1) separated children according to SIBO via 6 metabolites that had a VIP-score > 2 (Figure 1C2). The concentrations of glycolic acid and mannose were higher in SIBO-positive children, whereas the concentrations of valine, p-cresylsulphate (PCS), butyrate and adipic acid were higher in SIBO-negative children.

**Figure 2:**
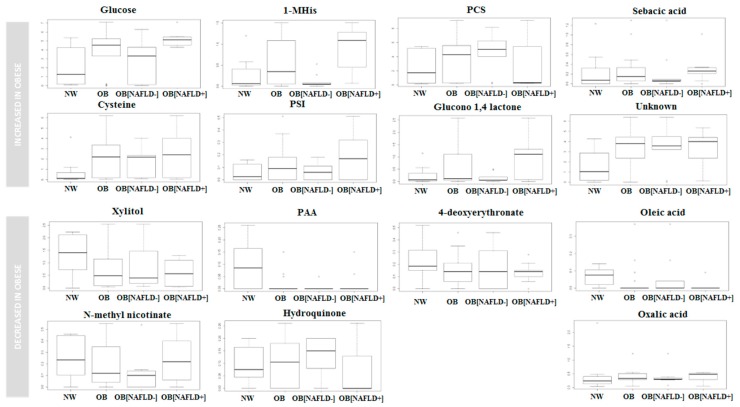
Box and Whisker plot of the VIP metabolites in the cohort of patients. Boxes represent normal weight controls (NW) *N =* 14; all the obese children (OB), *N =* 22; obese children without fatty liver (OB[NAFLD−], *N =* 10), and obese children with fatty liver (OB[NAFLD+], *N =* 12). The vertical axis reports the log of the GCMS value of the normalized area of each metabolite. Abbreviations: Gas Chromatography Mass Spectrometry (GCMS), 1-Methyl histidine (1-MHis), Normal weight (NW), Obese (OB), Phenylacetic acid (PAA), P-cresylsulphate (PCS), Pseudouridine (PSI), with (NAFLD+) without (NAFLD−) NAFLD.

As shown in Figure 2, the concentration of PCS was higher in obese subjects, especially those without steatosis, whereas the mean concentrations of glucose, methyl histidine (1-MHis), sebacic acid, pseudouridine (PSI), glucono-1,4-lactone and cysteine were higher prevalently in obese children with steatosis. On the contrary, the mean concentrations of xylitol, 4-phenyl acetic acid, oleic acid, 4-deoxyerythronate and *N*-methyl nicotinate were lower in obese children. Notably, *N*-methyl nicotinate was particularly low in children without steatosis. Altogether, the network of urinary molecules participating in groups separation was characterized by lower levels of xylitol (*p <* 0.05) and phenyl acetic acid (*p <* 0.05) in obese vs. normal weight individuals. The levels of glucose (*p <* 0.05) and 1-MHist (*p <* 0.05) were significantly higher in children with liver involvement.

The enrichment pathway analysis of the selected metabolites is summarized in the metabolic systems map shown in Supplementary Figure S1. There is a definite interplay of several pathways involving methionine and cysteine; glucose and xylitol metabolism; tyrosine metabolism; vitamin B3 (nicotinate and nicotinamide) metabolism; and the pentose 6 phosphate pathway.

**Figure 3:**
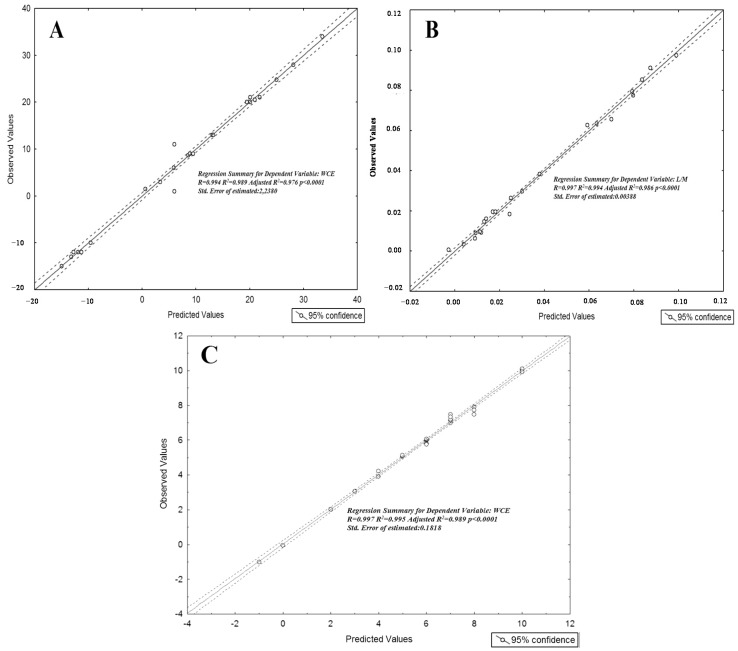
Stepwise multivariate linear regression analysis. (**A**) Correlation between observed vs. predicted value by the multivariate regression analysis using waist circumference centimeters exceeding the 90th percentile as dependent variable (*R*^2^ = 0.989); (**B**) Correlation between observed vs. predicted value by the multivariate regression analysis using lactulose/mannitol (L/M) ratio as dependent variable (*R*^2^ = 0.994); (**C**) Correlation between observed vs. predicted value by the multivariate regression analysis using KID-MED value as dependent variable (*R*^2^ = 0.995).

The stepwise regressions, correlating central adiposity, IP and the KidMed score with urinary metabolites, selected 12, 11 and 12 metabolites, respectively, with a statistically significant value (Table 2) and a good performance of prediction (Figure 3A–C).

**Figure 4:**
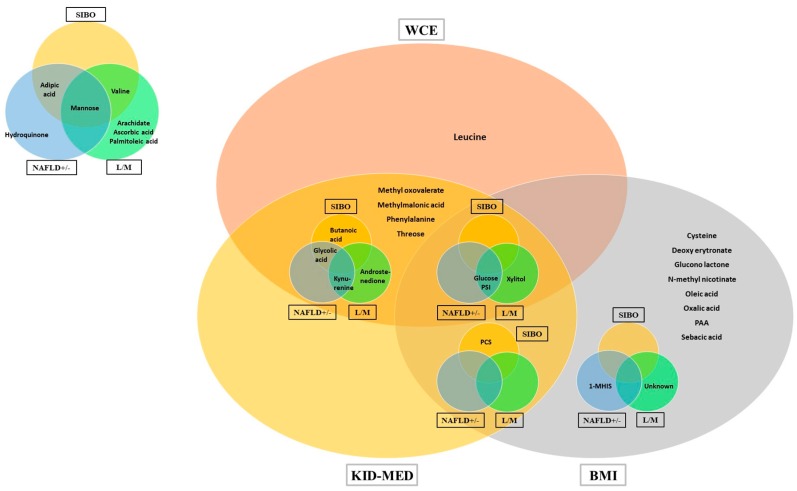
Venn diagram of the selected metabolites. Metabolites selected in the discriminant analysis (BMI, NAFLD and SIBO) and in the multivariate linear regression (WCE, L/M and KidMed). In the circle and oval overlaps, we represent the metabolites selected as VIP in more than one model. Only Glucose and pseudouridine (PSI) were common to all six models. Abbreviations: BMI (Body Mass Index), L/M (Lactulose to Mannitol ratio), (PSI) Pseudouridine, SIBO (Small Intestine Bacterial Overweight), WCE (centimeters exceeding 90th percentile of Waist Circumference), NAFLD (non-alcoholic fatty liver disease).

All metabolites associated with BMI, visceral obesity, fatty liver, intestinal permeability, SIBO and diet are summarized in a Venn diagram (Figure 4), which highlights the complex interaction of individual or grouped metabolites among these variously aggregated parameters.

## 4. Discussion

Our findings suggest that several metabolites and metabolic pathways contribute to a complex metabolic fingerprint of BMI, obesity, visceral obesity, and obesity related NAFLD. Moreover, these metabolic pathways appear to reflect the GLA alterations examined herein, i.e., intestinal permeability, SIBO and diet preferences. Some of these metabolites were easily predictable on the basis of obesity pathophisiology whereas others were not.

### 4.1. Obesity

The network of urinary molecules separating the lean and obese groups was characterized by lower levels of xylitol and PAA in obese individuals. Xylitol, a five-carbon sugar alcohol, naturally found in many types of fruits and vegetables, is not endogenously produced by humans and may thus reflect the type of diet. Xylitol has been linked to a healthy diet and considered beneficial in preventing the development of obesity and metabolic abnormalities in rats with diet-induced obesity [31]. In our study it appears to be associated with a protective effect against obesity-related liver damage since it was increased in NW patients.

Similar to xylitol, also urinary PAA in obese children was lower than in NW subjects. PAA is synthesized from the amino acid phenylalanine (PA) via phenylpyruvate. Plant secondary metabolites such as phenolic acids are generally associated with beneficial effects for human health [32], and as for xylitol, low urinary levels may reflect unhealthy diets characterized by a low intake of plant fibers. Moreover, PA urinary level has been linked also to 2-phenyletilamine metabolism, mainly related to monoamine oxidase activity. 2-phenylethylamine is an “endogenous amphetamine” that modulates central adrenergic functions, and low urinary phenyl acetate levels have been indicated as a marker of depression [33]. Notably the links among urinary metabolites, depression and pediatric obesity are currently much debated [34]. Our finding of high urinary levels of glucose in obese children is in line with the data previously reported in obese children [14,35,36,37] and adults [38]. This finding, which is not surprising from a pathological viewpoint given the higher incidence of insulin resistance in these individuals [39], is not trivial from a bioinformatics perspective, and can therefore be considered plausible evidence of the correctness of the classification model of our study.

### 4.2. NAFLD and Gut-Liver Axis Disturbances

The urinary concentration of methyl histidine, which had previously been associated with BMI in obese subjects [38], was increased in our patients, particularly in those with NAFLD. Regarding methyl histidine, this might reflect a more westernized diet, since it derives by enzyme (carnosinase) conversion mainly from the anserine of dietary meat sources. 1-methylhistidinuria may originate also from increased oxidation in skeletal muscle, reasonably eased by a reduction in the body’s antioxidants pool, e.g., by alpha-tocopherol deficiency, a condition commonly reported in pediatric obesity-related NAFLD [11,20,39]. Increased 1-MHis excretion in our obese children with NAFLD is in line with the low urinary amounts of xylitol observed in this category.

Urinary PSI level, which mainly originates from ribosomal and transfer RNA degradation, may reflect RNA turnover [40,41] and hence, like 1-MHis, is a measure of protein turnover. These metabolic processes, again, could be targeted by oxidative DNA stress in pediatric obesity and obesity-related NAFLD, in conditions such as high dietary energy intake [42].

Increased IP, gut microbiota dysbiosis, and SIBO have been associated with the severity of obesity-related liver damage [3,5,43,44]. Therefore, it is not surprising that a large percentage of our obese children with NAFLD showed signals of metabolome signature associated with gut-liver axis malfunctioning. In our study, urinary PCS, an intestinal microbial metabolite deriving from the secondary metabolism of p-cresol [45], was increased in obese children without NAFLD and correlated negatively with the presence of SIBO. Patel et al. [46] reported a significant reduction of PCS excretion in vegetarians associated with a 69% higher fiber intake and a 25% lower meat protein intake. This finding corroborates the inverse correlation we found (R = 0.49 *p <* 0.01) between PCS and the meat-intake-correlated metabolite 1-MHis.

In addition to histidine and phenylalanine metabolism, other amino acid metabolic pathways have been reported to be involved in obesity and GLA dysfunction [47]. Our results show that BCAA and/or their metabolites correlated with excess of visceral fat (leucine and oxo-valerate), increased IP and SIBO (valine metabolites). These findings are in keeping with recent reports that high concentrations of BCAAs in diet can induce insulin resistance and increased gluconeogenesis [36,48] contributing to upregulation of the TCA cycle, and the higher serum concentrations of its related catabolites in obese patients.

The high urinary cysteine concentration in our study cohort is consistent with the data reported in plasma of obese children [49] and adults [50] with NAFLD.

Finally, both N-methyl nicotinate and hydroquinone (HQ) urinary levels tended to be associated with a possible beneficial effect against liver steatosis, already signaled in adult NAFLD [51], in keeping with a cytoprotective role [52].

### 4.3. Study Limitations

Our study has a number of avoidable and unavoidable limitations. One is that liver involvement was not based on histology but only on the presence of US fatty liver and hypertransaminasemia. Ultrasound, at least in pediatrics, is easier, less expensive, non-invasive, quicker, and the only ethical option in normal weight subjects vs. liver biopsy which however remains clearly the gold standard diagnostic test [50].

In our study it emerged that transaminases values progressively increased from normal weight to obese subjects without and with US fatty liver, respectively. However, we did not consider sufficient to label as being affected by obesity related liver damage based on transaminase values only without concurrent US bright liver. In fact, hypertransaminasemia in obese population has been reported to be part of the signature of liver metabolic perturbations at the amino acid and Krebs cycle levels rather than the result of hepatocyte lysis alone [53]. Although the concordance of biochemical and US parameters cannot completely replace biopsy, in our opinion it was at least more likely to appropriately characterize each patients’ cohort.

Another limitation is that, given the pilot nature of the study, we were unable to exclude also the effects of physical exercise, gender and age. However, the variability of our children age was reassuringly low and mean age was comparable in all groups. Finally, although our metabolite platform enabled us to acquire profiles in a manner less biased than targeted methods, it did not enable the inclusion/identification of some highly specific analytes or an absolute quantification. However, our definite results in general indicate that a supervised model such as PLS-DA can overcome these confounders and allow a precise allocation of studied populations.

## 5. Conclusions

In summary, our pilot study has identified a complex network of urinary molecules that appear to be correlated with clinical phenotype, setting apart normal weight and obese children, and distinguishing between those with and without liver involvement, based also on the characterization of their GLA function. Individual or grouped metabolites interact with anthropometrics and variously aggregated GLA parameters.

Large functional and prospective studies, including clinical trials, are needed now to verify our preliminary results. Such studies will contribute to shed further insights into these metabolite profiles, and determine whether some of them are biomarkers or actual mediators of metabolic disease, and whether they can serve also as indicators of diet adherence [54]. A better understanding of the role of these biomarkers in obesity and obesity-related NAFLD pathogenesis might finally lead to novel targets for therapeutic/preventive intervention.

## Figures and Tables

**Table 1:** Characteristics of the study population.

Parameter	Control (NW) (*N =* 14)	Obese without NAFLD (*N =* 10)	Obese with NAFLD (*N =* 12)	All Obese (*N =* 22)
Gender (F/M)	5/9	5/5	4/8	9/13
Age (years)	11.25 ± 2.26	11.43 ± 2.32	11.66 ± 2.03	11.55 ± 2.12
Weight (kg)	37.48 ± 10.39	63.23 ± 17.97 *	65.05 ± 20.34 *	64.18 ± 18.79 *
Height (cm)	145.58 ± 15.21	150.71 ± 16.27	151.11 ± 15.62	150.92 ± 15.53
BMI (kg/cm^2^)	17.27 ± 2.09	27.46 ± 4.37 *	27.78 ± 4.93 *	27.63 ± 4.56 *
BMI Percentile	43.25 ± 25.28	98.60 ± 0.70 *	98.36 ± 0.92 *	98.48 ± 0.81 *
SDS BMI	−0.14 ± 0.82	2.50 ± 0.44 *	2.47 ± 0.50 *	2.48 ± 0.46 *
Waist Circumference (cm)	62.83 ± 8.61	81.36 ± 10.74 *	84.72 ± 11.24 *	83.12 ± 10.87 *
Waist Circumference percentile	35.83 ± 29.14	88.50 ± 4.74 *	88.64 ± 4.52 *	88.57 ± 4.51 *
Cm exceeding 90th percentile Waist Circumference	−7.69 ± 5.62	11.86 ± 6.83 *	14.26 ± 9.87 *	13.12 ± 8.44 *
WHtR	0.43 ± 0.04	0.54 ± 0.05 *	0.56 ± 0.06 *	0.55 ± 0.05 *
Hips Circumference (cm)	73.00 ± 11.21	93.75 ± 9.60 *	95.04 ± 14.16 *	94.42 ± 11.92 *
Systolic Pressure (mm Hg)	95.33 ± 12.63	111.00 ± 8.10 *	119.36 ± 12.63 *	115.38 ± 11.92 *
Systolic Pressure percentile	26.67 ± 29.61	62.30 ± 23.94 *	79.55 ± 21.40 *	71.33 ± 23.76 *
Systolic Pressure SDS	−0.83 ± 1.18	0.42 ± 0.80 *	1.20 ± 1.04 *	0.83 ± 1.00 *
Diastolic Pressure (mm Hg)	66.67 ± 7.78	69.10 ± 7.62	61.91 ± 8.54	65.52 ± 8.81
Diastolic Pressure percentile	65.42 ± 18.30	69.10 ± 22.01	50.45 ± 23.67	59.33 ± 24.27
Diastolic Pressure SDS	0.47 ± 0.63	0.62 ± 0.74	0.00 ± 0.66	0.30 ± 0.75
Glycaemia (mg/dL)	78.25 ± 19.14	85.63 ± 8.48 *	90.54 ± 8.20 *	88.23 ± 8.45 *
Insulinemia (mU/mL)	11.52 ± 4.32	12.24 ± 6.19	18.56 ± 12.15	16.13 ± 10.45
HOMA-IR [(Glu *Ins)/405]	2.24 ± 1.12	2.87 ± 1.51	3.70 ± 2.50	3.31 ± 2.07
ALT (U/L)	15.83 ± 8.65	20.88 ± 4.02	49.80 ± 15.75 *	36.19 ± 18.78 *
AST (U/L)	25.25 ± 2.96	25.36 ± 8.38	31.56 ± 14.52	28.46 ± 16.76
Intestinal Permebility (L/M ratio)	0.0158 ± 0.0121	0.0209 ± 0.0214	0.0630 ± 0.0328 *	0.0420 ± 0.0392 *
SIBO (n/total) (% positive pts)	0/14 (0%)	5/10 (50.0%)	7/12 (58.3%)	12/22 (54.5%) *
KIDMED score	7.88 + 1.69	5.00 + 2.23 *	5.23 + 2.74 *	5.12 + 2.52 *

Most of the values are expressed as means ± 1 standard deviation. * Asterisk indicates a statistically significant difference (*p <* 0.003) from the Control group. No statistically significant differences were observed between obese children with and without NAFLD. Abbreviations: ALT (Alanine Transaminase, normal upper value threshold <26 U/L boys; 22 U/L girls), AST (Aspartate Transaminase), BMI (Body Mass Index), HOMA-IR (Homeostatic Model Assessment – Insulin Resistance), L/M (Lactulose to Mannitol ratio), NW (Normal Weight), SDS (Standard Deviation), SIBO (Small Intestine Bacterial Overgrowth), wo (without), WtHR (Waist to Height Ratio).

**Table 2:** Stepwise multivariate regression for WCE, L/M and KID-MED as dependent variable.

		Beta	Std.Err.	*p*-Value
WCE	Leucine	0.883	0.054	<0.001
	Glucose	0.857	0.057	<0.001
	Xylitol	0.174	0.046	0.003
	Glycolic acid	0.491	0.049	<0.001
	Methyloxovalerate	−0.669	0.068	<0.001
	Pseudouridin	0.204	0.081	0.03
	Methylmalonic acid	−0.263	0.050	<0.001
	Androstenedione	0.355	0.060	<0.001
	Butanoic acid	−0.206	0.054	<0.001
	Phenylalanine	0.154	0.069	0.04
	Kynurenine	−0.089	0.052	0.01
	Threose	0.045	0.044	0.02
L/M	Glucose	0.402	0.040	<0.001
	Mannose	−1.083	0.046	<0.001
	Arachidate	−0.449	0.043	<0.001
	Palmitoleic acid	0.202	0.044	0.001
	Ascorbic acid	0.672	0.041	<0.001
	Xylose	−0.720	0.065	<0.001
	Kynurenine	−0.207	0.037	<0.001
	Pseudouridine	−0.017	0.040	0.04
	Valine	0.317	0.053	<0.001
	Androstenedione	0.357	0.061	<0.001
	Unknown	−0.213	0.044	<0.001
KID-MED	Methyloxovalerate	−1.106	0.033	<0.001
	Methylmalonic acid	−0.459	0.027	<0.001
	Butanoic acid	−0.347	0.035	<0.001
	Kynurenine	−0.145	0.027	<0.001
	p-cresylsulphate	−0.139	0.030	<0.001
	Threose	0.082	0.032	0.024
	Phenylalanine	0.100	0.031	0.008
	Xylitol	0.117	0.036	0.007
	Pseudouridin	0.177	0.024	<0.001
	Androstenedione	0.326	0.028	<0.001
	Glycolic acid	0.438	0.029	<0.001
	Glucose	1.015	0.046	<0.001

Abbreviations: L/M (Lactulose to Mannitol ratio), WCE (centimeters exceeding 90th percentile of Waist circumference).

## Supplementary Materials

The following are available online at www.mdpi.com/2072-6643/9/5/485/s1, Figure S1: Metabolic systems map summarizing the shortest route that may explain the interactions among the 27 selected metabolites. Table S1: Daily dietary nutrients assessment through 24 h recall.
