# Supplementary material for: Urinary Metabolomics in Pediatric Obesity and NAFLD Identifies Metabolic Pathways/Metabolites Related to Dietary Habits and Gut-Liver Axis Perturbations
Source: Nutrients. 2017 May 11;9(5):485. doi: 10.3390/nu9050485 (PMC5452215; doi:10.3390/nu9050485)
Supplement: Supplementary file 1 [file nutrients-09-00485-s001.pdf]

Supplementary

Figure S1

Metabolic systems map summarizing the shortest route that may explain the interactions among the 27 selected metabolites.

There is a clear interplay of several pathways involving: Androgen and estrogen biosynthesis and metabolism; Biopterin metabolism; Butanoate metabolism; C21-steroid hormone biosynthesis and metabolism; Fructose and mannose metabolism; Galactose metabolism; Glycine, serine, alanine and threonine metabolism; Methionine and cysteine metabolism; Pentose phosphate pathway; Tryptophan metabolism; Tyrosine metabolism; Urea cycle and metabolism of arginine, proline, glutamate, aspartate and asparagine; Valine, leucine and isoleucine degradation; Vitamin B3 (nicotinate and nicotinamide) metabolism; Vitamin B5 - CoA biosynthesis from pantothenate.

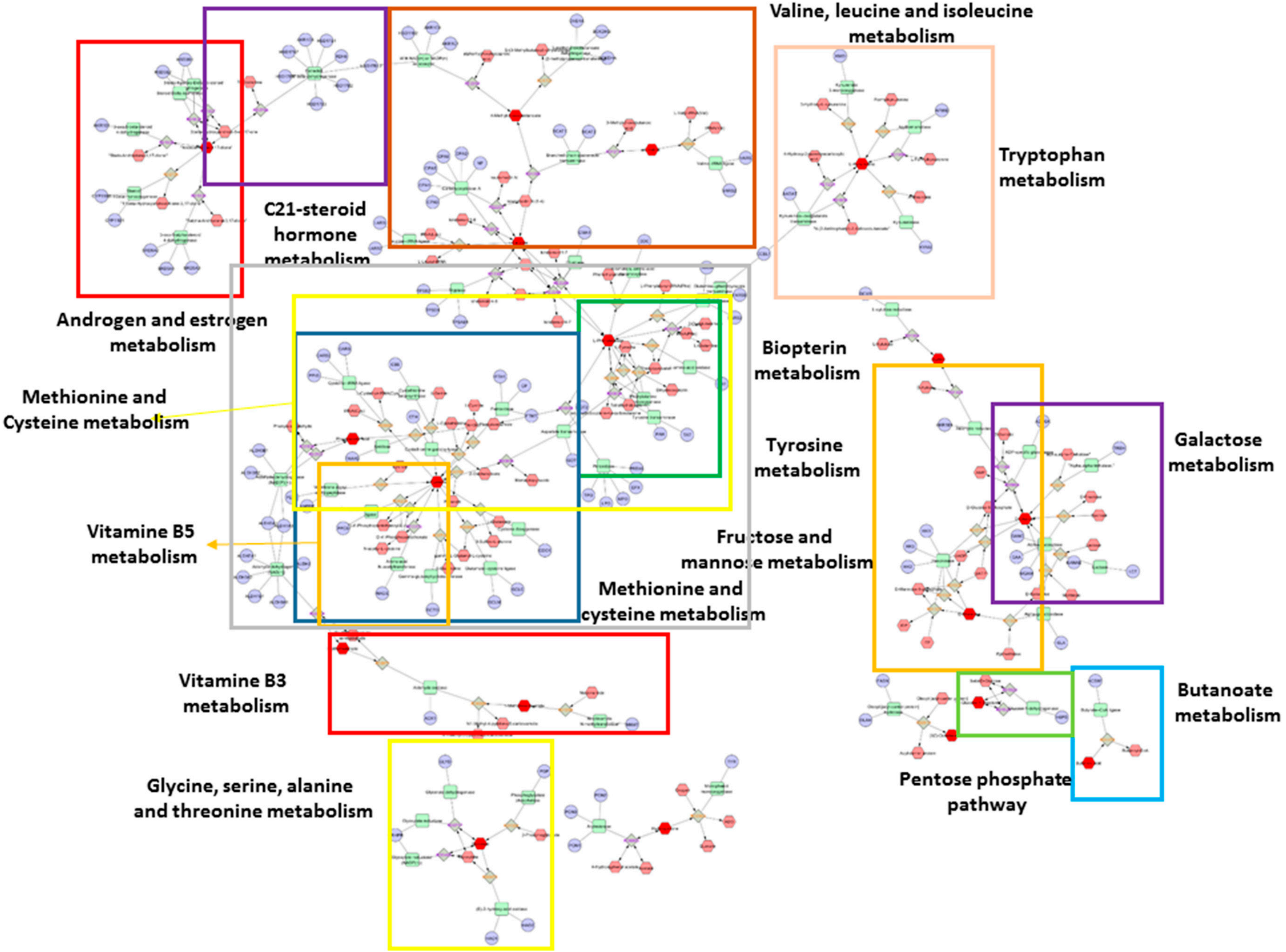

Valine, leucine and isoleucine metabolism
Tryptophan metabolism
C21-steroid hormone metabolism
Androgen and estrogen metabolism
Biopterin metabolism
Methionine and Cysteine metabolism
Tyrosine metabolism
Galactose metabolism
Vitamine B5 metabolism
Fructose and mannose metabolism
Methionine and cysteine metabolism
Vitamine B3 metabolism
Butanoate metabolism
Pentose phosphate pathway
Glycine, serine, alanine and threonine metabolism

Table S1. Daily dietary nutrients assessment through 24 hrs recall

		**NW**	**OB[NAFLD-]**	**OB[NAFLD+]**
Energy intake	*Kcal/die*	1330,3±314,4	1568,4±295,6	1739,6±505,4*
Total carbohydrates	*g/die*	180,8±52,8	184,8±63,4	177,3±67,7
	%	50,6±8,2	43,8±7,1*	38,3±7,8*
Simple Sugars	*g/die*	21,2±4,2	43,3±27,3	41,2±17,7
	%	6,4±1,1	10,3±5,9	9,0±3,3
Total fats	*g/die*	50,1±17,3	70,7±12,8*	83,3±21,4*
	%	33,2±9,2	41,0±6,3*	43,7±6,0*
Saturated fats	*g/die*	8,9±5,8	17,7±7,3	18,4±6,4*
	%	11,7±6,0	10,3±3,7	9,9±3,2
Total fibers	*d/die*	9,0±4,1	10,8±3,2	11,3±5,4
Total proteins	*g/die*	50,7±13,3	57,8±11,3	78,4±35,8*
	%	15,0±3,6	15,3±4,2	18,1±4,7
Cholesterol	*mg/die*	152,9±124,9	159,8±82,6	267,0±186,6
Sodium	*mg/die*	726,3±505,2	1452,7±746,3	1781,1±955,7
Fructose	*mg/die*	6,9±4,2	9,9±4,7	19,7±17,0*§

Abbreviations: (NAFLD) non alcoholic fatty liver disease; (NW), normal weight/controls; (OB[NAFLD-]) obese without NAFLD; (OB[NAFLD+]) obese with NAFLD

Statistics: Values are expressed as means ± 1 standard deviation. * asterisk indicates a statistically significant difference (p<0.05) from the Control group (NW), § symbol indicates a statistically significant difference (p<0.05) from the obese without NAFLD (OB[NAFLD-]).
